# Identifying Practice Gaps Among Otolaryngology Providers for the Treatment of Chronic Cough

**DOI:** 10.1002/oto2.143

**Published:** 2024-05-15

**Authors:** Natalie L. Demirjian, Austin Lever, Helena Yip

**Affiliations:** ^1^ University of Arizona College of Medicine–Tucson Tucson Arizona USA; ^2^ Department of Otolaryngology–Head and Neck Surgery University of Arizona College of Medicine–Tucson, Banner University Medical Center Tucson Arizona USA

**Keywords:** chronic cough, laryngeal hypersensitivity, neuromodulators, speech therapy

## Abstract

**Objective:**

Increasing evidence over the last decade suggests that many cases of unexplained chronic cough (UCC) have a neurogenic etiology, with laryngeal hypersensitivity (LH) being identified as a key mechanism. Official guidelines since 2015 have adopted use of neuromodulators and adjuvant speech therapy as a result, but historically implementation of guidelines is slow. Our survey aimed to investigate gaps in diagnosis and management practices of otolaryngology providers in caring for patients with UCC.

**Study Design:**

Cross‐sectional study.

**Setting:**

Survey.

**Methods:**

12‐item survey was distributed to 110 otolaryngology practitioners experienced in diagnosis and treatment of chronic cough at a regional otolaryngology continuing education conference. Statistical analysis included Kendall's Tau Rank Correlation to measure the ordinal association between responses to questions, and Fisher's exact test to determine if there were associations between responses and years of career experience.

**Results:**

Forty eligible respondents underwent subsequent analysis. There was no association between frequency of identifying LH as a primary etiology and use of neuromodulators (*τ* = 0.23, *P* = .10). However, there was a significant correlation between LH and referrals to speech therapy (*τ* = 0.27, *P* = .05). Fisher's exact test did not reveal any significant differences among any responses based on practitioner experience.

**Conclusion:**

Our results indicate a possible disparity in treatment of UCC with neuromodulators and the utilization of speech therapy despite guideline recommendations advocating for neuromodulators with adjuvant speech therapy. Further research with larger sample sizes and more specific inquiries is necessary to elucidate this association and control for any regional differences.

Chronic neurogenic cough is an issue that affects nearly 11% of population worldwide and carries with it significant morbidity and impairs quality of life.^1^, ^2^ These patients can face sleep disruption, social isolation, depression, and loss of employment due to persistent cough symptoms. Chronic neurogenic cough is defined as a cough lasting for longer than 8 weeks of unknown cause and refractory to standard therapy.^1^, ^2^ These patients typically undergo extensive medical workup and treatment across multiple subspecialties without improvements in their symptoms.^1^, ^2^, ^3^

Increasing evidence over the last decade suggests that many causes of unexplained or refractory chronic cough (UCC) are due to a sensory neuropathy in the hypopharynx and larynx, leading to laryngeal hypersensitivity (LH) being identified as a key mechanism.^4^, ^5^, ^6^ Once other common etiologies of cough such as asthma, gastroesophageal reflux disease, and medication side effects have been ruled out as contributing factors, treatment can then be initiated which targets the neurogenic pathways leading to cough symptoms.^1^ As of 2015, official guidelines from the American College of Chest Physicians (CHEST) have recommended the use of gabapentin and adjuvant speech therapy for treatment.^1^

The use of specific neuromodulators, specifically the use of gabapentin, pregabalin, and tricyclic antidepressants have all shown efficacy in treating cough symptoms related to neurogenic cough.^7^, ^8^, ^9^, ^10^ More recently, procedural therapy with the use of superior laryngeal nerve blocks, vocal cord augmentation, and laryngeal botulinum toxin injection have also been shown to be effective in improvement of symptoms.^11^, ^12^, ^13^ Cough suppression therapy administered through speech‐language pathologists is also an effective tool alone or in combination with pharmacotherapy or procedural therapy.^14^ Despite the official guidelines, evidence, and the array of treatments available to target the neuropathic etiology of cough symptoms, there is a heterogeneity in diagnosis and treatments practices among otolaryngologists.^15^

Delay in the implementation of guidelines in clinical practice has been studied, and it has been found to take an average of 17 years before there is a change in clinical practice despite changes in guidelines and advances in research.^15^ It is important to note, however, that technology and advances in communication have risen exponentially since the execution of many of these studies, and accordingly there has been an increase in the accessibility of any ongoing research and recommendations due to developments of technology.

The goal of this study is to investigate the presence of any gaps in the diagnosis and management practices among otolaryngology providers who care for patients with chronic cough.

## Materials and Methods

This study was designed as physical survey which aimed to capture the treatment practices of unexplained chronic cough by local otolaryngologists and was developed by a fellowship‐trained laryngologist. This study was approved by the University of Arizona institutional review board (application #STUDY00003289).

### The Survey

The principal investigator of this study, whom is a fellowship‐trained laryngologist, drafted and tested the survey. The survey was comprised of 12‐items and had an estimated completion time of less than 5 minutes. Data collected included general demographics and qualifications such as degree, length of practice, and type of practice. Surveys were kept anonymous by excluding any identifying information such as name or institution of practice. Nine items concerned the diagnosis and treatment of chronic cough focused on the following domains: laryngopharyngeal reflux (LPR), use of proton pump inhibitors (PPIs), LH, use of neuromodulators, use of speech therapy, and performance of superior laryngeal nerve blocks as well as laryngeal Botox injections (Table 1). Response options on cough‐related questions required participants to rate agreement or frequency using a Likert scale of 5 ordered response levels with an opportunity to respond to 1 short answer question based a prior response.

**Table 1:** 12‐Item Survey With Associated Response Rates

Background	
(1) I'm a:	
MD/DO	31/40 (77.5%)
Nurse Practitioner	6/40 (15%)
Physician Assistant	3/40 (7.5%)
(2) Length of Practice^a^:	
In training	2/40 (5%)
0‐5 years	9/40 (22.5%)
5‐10 years	1/40 (2.5%)
10‐15 years	5/40 (12.5%)
15+ years	23/40 (57.5%)
(3) My practice is:	
Comprehensive	35/40 (87.5%)
Subspecialty‐oriented	5/40 (12.5%)

Thinking about your evaluation and management of patients with chronic cough over the past 5 years, answer the following questions about the etiology and your treatments:	
(4) I see chronic cough:	
Almost never	1/40 (2.5%)
Occasionally	8/40 (20%)
Sometimes	6/40 (15%)
Frequently	21/40 (52.5%)
All the time	4/40 (10%)
(5) Laryngopharyngeal reflux was found as the primary etiology of chronic cough:	
Strongly disagree	2/40 (5%)
Somewhat disagree	4/40 (10%)
Neutral	5/40 (12.5%)
Somewhat agree	18/40 (45%)
Strongly agree	11/40 (27.5%)
(6) I have used proton pump inhibitors (PPIs) as a first line therapy in the treatment of chronic cough:	
Strongly disagree	0/40 (0%)
Somewhat disagree	1/40 (2.5%)
Neutral	4/40 (10%)
Somewhat agree	20/40 (50%)
Strongly agree	15/40 (37.5%)
(7) Laryngeal hypersensitivity was found as the primary etiology of chronic cough:	
Strongly disagree	2/39 (5.1%)
Somewhat disagree	5/39 (12.8%)
Neutral	11/39 (28.2%)
Somewhat agree	16/39 (41.0%)
Strongly agree	5/39 (12.8%)
(8) Do you use gabapentin or amitriptyline in the treatment of chronic cough?:	
Almost never	17/40 (42.5%)
Occasionally	6/40 (15%)
Sometimes	11/40 (27.5%)
Frequently	5/40 (12.5%)
All the time	1/40 (2.5%)
(9) If yes, what is the preferred maximal dose you prescribe?^b^	16/40 (40%)
(10) Do you refer patients to speech therapy for cough suppression therapy?:	
Almost never	19/39 (48.7%)
Occasionally	6/39 (15.4%)
Sometimes	11/39 (28.2%)
Frequently	3/39 (7.7%)
All the time	0/39 (0%)
(11) Do you perform superior laryngeal nerve blocks?:	
Almost never	35/39 (89.7%)
Occasionally	0/39 (0%)
Sometimes	4/39 (10.3%)
Frequently	0/39 (0%)
All the time	0/39 (0%)
(12) Do you perform laryngeal Botox injections?:	
Almost never	37/39 (94.9%)
Occasionally	1/39 (2.6%)
Sometimes	1/39 (2.6%)
Frequently	0/39 (0%)
All the time	0/39 (0%)

All data are presented as numerators and denominators with percentages in parentheses.

^a^ Early career was defined as <15 years of practice.

^b^ Hidden question based on previous answer that gave the option to “write‐in” a response.

### Survey Design

The survey was physically distributed to 110 otolaryngology practitioners at a continuing medical education conference hosted by our institution after a brief explanation of the study. Participation in the survey was voluntary, and survey forms were collected from participants by the principal investigator of this study before the conclusion of the conference. Survey data was then manually input into REDCap^TM^ (Research Electronic Data Capture) hosted by our institution for secure data storage. Once all data was stored in REDCap^TM^, the physical surveys were securely discarded.

### Data Analysis

Descriptive analysis included percent distribution of responses, and Kendall's Tau Rank Correlation (Tau, *τ*) was performed to measure the ordinal association between responses to questions. Fisher's exact test and Cramer's *V* were also performed to determine if there were associations between length of career experience and responses as well as frequency of chronic cough and responses. Length of career experience was divided into 2 categories, early career and late career, where early career was defined as <15 years of practice experience while late career was defined as >15 years of experience. Frequency of chronic cough in practice was divided into 2 broad categories. The nonfrequent group was defined as those who responded with “almost never,” “occasionally,” and “sometimes” while and frequent group was comprised of those who responded with “frequently” or “all the time.” Fisher's Exact tests regarding questions pertaining to level of agreement were divided into 2 categories: nonagreement (ie, those who responded with “strongly disagree,” “somewhat disagree,” or “neutral”) and agreement (ie, those who responded with “somewhat agree” or “strongly agree”).

Statistical analysis was performed using MATLAB version R2023b.

## Results

**Figure 1:**
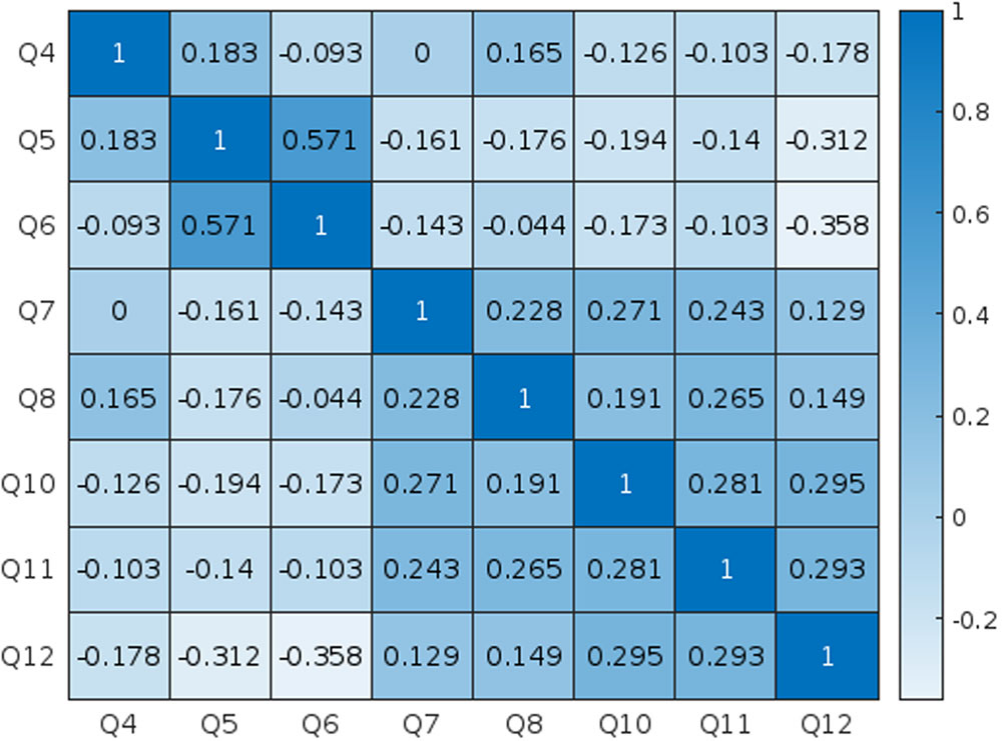
Heatmap of Kendall's Tau coefficients between survey questions. Heatmap of Kendall's Tau Rank Coefficients between each survey question, except for background questions (Q1‐3).

Of the 110 surveys distributed, there were 42 respondents to the survey. Only otolaryngology providers (MD/DO, Nurse Practitioner, or Physician Assistant) with direct experience in the diagnosis and management of chronic cough were included for analysis; thus, 2 surveys were excluded as these did not meet criteria. One item on the survey was discarded prior to statistical analysis due to poor clarity. A summary of the survey results is provided in Table 1. A heat map to summarize Kendall's Tau correlation coefficients between survey questions is depicted in Figure 1 with associated *P*‐values in Table 2.

**Table 2:** Summary of Kendall's Tau Coefficient Values and Associated *P*‐Scores Between Survey Questions. Question numbers (Q#) Correspond to Numbered Questions Found in Table 1

		Q4	Q5	Q6	Q7	Q8	Q10	Q11	Q12
Q4	Correlation	1.00	0.18	−0.09	0.00	0.17	−0.13	−0.10	−0.18
	*P* value	0.00	0.19	0.53	1.00	0.24	0.38	0.51	0.25
Q5	Correlation	0.18	1.00	0.57	−0.16	−0.18	−0.19	−0.14	−0.31
	*P* value	0.19	0.00	<0.001	0.25	0.21	0.17	0.36	0.04
Q6	Correlation	−0.09	0.57	1.00	−0.14	−0.04	−0.17	−0.10	−0.36
	*P* value	0.53	<0.001	0.00	0.32	0.77	0.24	0.52	0.02
Q7	Correlation	0.00	−0.16	−0.14	1.00	0.23	0.27	0.24	0.13
	*P* value	1.00	0.25	0.32	0.00	0.10	0.05	0.11	0.40
Q8	Correlation	0.17	−0.18	−0.04	0.23	1.00	0.19	0.27	0.15
	*P* value	0.24	0.21	0.77	0.10	0.00	0.18	0.08	0.33
Q10	Correlation	−0.13	−0.19	−0.17	0.27	0.19	1.00	0.28	0.30
	*P* value	0.38	0.17	0.24	0.05	0.18	0.00	0.07	0.05
Q11	Correlation	−0.10	−0.14	−0.10	0.24	0.27	0.28	1.00	0.29
	*P* value	0.51	0.36	0.52	0.11	0.08	0.07	0.00	0.08
Q12	Correlation	−0.18	−0.31	−0.36	0.13	0.15	0.30	0.29	1.00
	*P* value	0.25	0.04	0.02	0.40	0.33	0.05	0.08	0.00

Of the 40 eligible respondents that underwent subsequent analysis, 31 (77.5%) held the degree of MD or DO, 6 (15%) were nurse practitioners, and 3 were physician assistants (7.5%). Majority of respondents (57.5%) were considered late career with greater than or equal to 15 years of practice experience in the field. Thirty‐five out of 40 respondents (87.5%) considered their practice to be comprehensive rather than subspecialty oriented (12.5%).

Regarding the frequency with which respondents have seen chronic cough in their practice over the past 5 years, majority of respondents responded “frequently,” followed by “occasionally” (52.5% and 20%, respectively). Fisher's exact test did not reveal any significant differences among any of the survey questions based on length of practice experience.

When asked the level of agreement with the statement that LPR was found as a primary etiology of chronic cough, nearly half of patients responded “somewhat agree” (45%) while 27.5% responded “strongly agree.” Fisher's Exact test did not find any significant relationship between length of practice (early career versus late career) and responses to the aforementioned LPR question (*P* = .39 and *P* = .31, respectively). Cramer's *V* was equal to 0.31 for the same data, indicative of moderate effect size.

Fifty percent of respondents noted “somewhat agree” to the statement “I have used PPIs as a first‐line therapy in the treatment of chronic cough,” and 37.5% noted “strongly agree.” Fisher's exact test revealed a significant association between those who indicated agreement with LPR as a primary diagnosis and those indicated agreement with use of PPIs in their practice (*P* = .00007). Kendell's Tau coefficient revealed a positive association between frequency of identifying LPR as the primary etiology of chronic cough and use of PPIs as first‐line therapy (*τ* = 0.57, *P* < .001).

One respondent did not complete the question pertaining to level of agreement with the statement “laryngeal hypersensitivity was found as the primary etiology of chronic cough.” As a result, this individual's responses were excluded from analysis with any computations that utilized data from this question. The most frequent answer to this question among the available respondents was “somewhat agreed” at 16 out of 39 (41%). Second most frequent response was “neutral” with 11 out of 39 (29.2%). Fisher's Exact test did not find a significant relationship between length of practice and responses (*P* = .845).

When asked the frequency with which they use neuromodulators for treatment of chronic cough, 17 out of 40 respondents replied “almost never” while 11 out of 40 reported “sometimes.” Fischer's exact testing did not reveal any association among these responses with length of training. Kendell's Tau coefficient investigating the relationship between those who identified LH as a primary etiology and frequency of prescribed neuromodulators was 0.23 with *P* = .10.

Frequency of speech therapy referrals for chronic cough suppression was also investigated. Among our survey responses, nearly half responded “almost never” (48.7%), followed by the response “sometimes” at 28.2%. Kendall's Tau coefficient was 0.27 (*P* = .05) for the association between those who found LH as a primary etiology of chronic cough more frequently and the frequency of speech therapy referrals.

89.7% and 94.9% of respondents replied “almost never” when asked the frequency with which they performed superior laryngeal nerve blocks and laryngeal Botox injections, respectively. Kendall's Tau coefficient between these questions and frequency of LH as a primary etiology of chronic cough was 0.24 and 0.13, respectively (*P* = .11 and *P* = .40, respectively).

## Discussion

The degree of significance between the frequency of identifying LPR as a primary etiology of chronic cough and frequency of PPI prescription (*P* < .01) was largely expected given the high prevalence of the condition and PPIs being widely accepted as an appropriate first‐line treatment for cough in the setting of suggestive history, physical findings, and diagnostic studies among otolaryngologists. Therefore, our results confirm appropriate use of these medications in accordance with guideline recommendations for chronic cough across otolaryngology providers regardless of career length.

Our results suggest a continued lack of implementation of guideline recommendations in the treatment of chronic cough among otolaryngology providers despite presence of these recommendations approaching almost a decade. Given the frequency with which respondents identify the disease in their respective clinical practices, our results are less suggestive of a knowledge gap on the topic of LH as an etiology of chronic cough, despite the relatively recent recognition of the topic among the scientific community.^5^ Moreover, there was not any significant differences on responses based on career experience—which might be expected with those earlier in their career demonstrating practices more consistent with guideline recommendations.

Rather, lack of any statistical significance for the association between those who frequently identified LH as a primary etiology and the frequency with which providers perform the guideline‐recommended treatments for chronic cough suggest a practice gap present between ability to diagnose LH and the execution of recommended treatment practices. Notably, the moderate association between frequency of LH as a chronic cough etiology and frequency of speech therapy referral, which approached near significance at *P* = .051, was relatively stronger than the relationship between LH as an etiology and frequency of neuromodulator prescription (*τ* = 0.23, *P* = .10). These results may suggest that providers are more inclined to recommend speech therapy to their patients with LH rather than prescribe neuromodulators in addition to speech therapy.

Of note, most of the providers who participated in this study were comprehensive otolaryngologists, and there was a lack of representation of laryngologists among our respondents. Our study is more applicable to the treatment practices of chronic cough among otolaryngology providers that are not focused on the larynx. Results pertaining to the evaluation and management practices among laryngologists may differ significantly from our results. For example, most of our respondents do not perform more specialized laryngeal procedures such as superior laryngeal nerve blocks and laryngeal Botox injections, 2 procedures more commonly performed by a dedicated laryngologist. Yet, it is important to note the relatively limited quantity of laryngologists across the country. Access to laryngologists may be more restricted in more rural areas of the United States, and treatment of chronic cough in those regions may rely on practices of the comprehensive otolaryngologists or even primary care physicians.

This study has several limitations: First, the survey design of this study inherently lends itself to nonresponse bias. While survey distribution at an in‐person conference helped to increase proportion of survey respondents compared to other survey studies, but our sample size remains relatively low overall, and therefore the statistical power, generalizability, precision, ability to perform subgroup analysis are reduced. Second, the survey was distributed at a regional continuing medical education conference for otolaryngology providers and is therefore primarily derived from health care professionals practicing in 1 region of the United States. This geographic influence further reduces the generalizability of our results. Third, the impact of patient‐centered factors was not evaluated in our survey and may play a role in our results; for example, patients may refuse pharmacological recommendations for a variety of reasons, or a provider's patient population may have more limited access to speech therapy resources geographically. Therefore, these factors could influence the responses gathered from the participating providers and is therefore a limitation of this study. Fourth, the survey lacked questions on whether the lack of use of neuromodulators or adjunct treatments was due to lack of buy in, or lack of awareness, or simply due to a workflow where these patients were referred to a laryngologist. Therefore, the survey cannot capture the full details of the providers' thoughts on the use of adjunct treatments to cough and cannot identify actual practice patterns as they might include referral to specialists. Further research should investigate the specific factors that may play a role in this practice gap between LH diagnosis and use of guideline recommendations for pharmacological intervention with neuromodulators such as gabapentin with adjuvant speech therapy for cough suppression.

## Conclusion

Our findings indicate that despite advancements in technology and increased access to information, implementation of CHEST guideline recommendations for the treatment of chronic cough remains slow. Otolaryngologist managing patients with chronic cough may be underutilizing neuromodulators and speech therapy. Further research with larger sample sizes and more specific inquiries is necessary to elucidate this association and control for any regional differences.

## Author Contributions

Helena Yip and Austin Lever developed and distributed surveys to participating otolaryngology providers. Natalie L. Demirjian assisted with data curation and visualization, as well as formal analysis with assistance of a statistician. Natalie L. Demirjian and Austin Lever drafted the manuscript, with support and supervision from Helena Yip who was responsible for conceptualization. All authors approved the manuscript prior to submission.

The authors declare that they had full access to all the data in this study and the authors take complete responsibility for the integrity of the data and the accuracy of the data analysis.

## Disclosures

### Competing interests

None.

### Funding source

None.
